# Comparison Between the Efficacy of EFR and CASL in the Treatment of Small Gastric Stromal Tumors: A Retrospective Study

**DOI:** 10.5152/tjg.2025.24461

**Published:** 2025-06-16

**Authors:** Liang Ye, Binbin Huang, Xiaoyuan Yi, Huaiyang Cai

**Affiliations:** 1 Department of Gastroenterology, Liuzhou People’s Hospital affiliated to Guangxi Medical University, Liuzhou, China; 2 Department of Gastroenterology, Affiliated Hospital of Guilin Medical University, Guilin, China

**Keywords:** Cap-aspiration lumpectomy, elderly, full-thickness resection, small gastric GISTs

## Abstract

**Background/Aims:**

This study aimed to assess the efficacy and safety of endoscopicfull-thickness resection (EFR) in comparison to cap-aspiration lumpectomy (CASL) for treating small gastrointestinal (GI) stromal tumors (GISTs).

**Materials and Methods:**

A retrospective analysis was carried out on the data from elderly patients (66 cases) with small gastric GISTs who were treated with EFR (41 cases) or CASL (25 cases). The study compared the clinical features, surgical conditions, intraoperative and postoperative complications, postoperative efficacy, economic benefits, and follow-up of small gastric GISTs in the EFR and CASL groups.

**Results:**

The mean operative time for the EFR group was longer than that for the CASL group [45.0 (32.5, 66.5) minutes versus 30.0 (20.0, 42.5) minutes]; the resection time in the EFR group was higher than that in the CASL group [30.0 (20.0, 50.5) versus 9.0 (6.5, 16.5) minutes]; the rate of utilization of hot hemostatic forceps in the EFR cohort was higher than that observed in the CASL cohort [75.6% (31/41) versus 12.0% (3/25)]; the postoperative course of antibiotics was longer in the EFR group than in the CASL group [(2.8 ± 2.0) d versus (1.0 ± 2.0) d]; and the hospitalization cost of the EFR group was extremely higher than that of the CASL group [(¥13 595.0 ± 2653.3) versus (¥11 209.0 ± 2458.9)].

**Conclusion:**

EFR and CASL are safe and effective in the treatment of small gastric GISTs, and CASL is more suitable for the treatment of small gastric GISTs located in the gastric fundus and body in elderly patients.

**Main Points**

- Gastric gastrointestinal stromal tumors (GISTs) are the most common tumors of stromal origin in the digestive tract.
- EFR and cap-aspiration lumpectomy (CASL) are more suitable for the treatment of small gastric GISTs.
- CASL results in shorter operative time, fewer postoperative inflammatory reactions, and lower medical costs compared with EFR.
- Cap-aspiration lumpectomy is a more appropriate treatment option for small gastric GISTs in elderly patients.

## Introduction

Gastrointestinal stromal tumors (GISTs) represent the most prevalent tumors of stromal origin within the digestive tract. However, gastric GISTs account for 60%.^1^ These conditions are most prevalent in the middle-aged and elderly population, with a more insidious onset of disease, and are more prone to metastasis or plasma membrane metastasis than in the young.^2^^,^^3^ The stromal tumors ≤ 2 cm are known as small GISTs, which are usually asymptomatic, have a high incidence, and those with a high nuclear schizophrenic image are also invasive. Currently, the optimal management of small gastric GISTs remains a topic of debate in the medical community, and there are no guidelines to clarify the treatment criteria for small gastric GISTs in elderly patients.^4^^,^^5^ In light of the financial implications of prolonged surveillance and the potential for tumor growth and metastasis, some experts believe that small stromal tumors should be resected as soon as they are found endoscopically.^6^ Laparoscopic surgery and traditional surgery are still the standard for the treatment of gastric GISTs, but in recent years, endoscopic treatment have also been gradually applied to gastric GISTs ≤ 5 cm.^7^ The treatment of GISTs is mainly surgical, but in recent years, laparoscopic surgery and endoscopic treatment have also been gradually applied to gastric GISTs ≤ 5 cm. Compared with surgical and laparoscopic procedures, endoscopic treatment offers several advantages, including being minimally invasive, having fewer complications, quicker postoperative recovery, and lower cost.^7^^-^^9^ Given the psychological and physical variances between elderly patients and their younger counterparts, endoscopic surgery is unquestionably a superior option for treating small gastric GISTs in elderly patients. Since most gastric GISTs originate from the lamina propria, grow intraluminally, and are located in the fundus, endoscopic full-thickness resection (EFR) and cap-aspiration lumpectomy (CASL) are more suitable for the treatment of small gastric GISTs. So far, the efficacy and safety of EFR and CASL in the treatment of small gastric GISTs in the elderly have seldom been documented in the literature. This study aims to compare the efficacy of the 2 surgical modalities in treating small gastric GISTs in elderly patients.

## Materials and Methods

### Study Population

This study retrospectively analyzed a total of 66 elderly patients who were diagnosed with small gastric GISTs and underwent endoscopic total resection from May 2022 to May 2023 at the affiliated Hospital of Guilin Medical University and the Liuzhou People’s Hospital affiliated to Guangxi Medical University. Inclusion criteria: (1) age ≥60 years (according to the WHO age classification standard); (2) postoperative histopathological diagnosis of gastric stromal tumor; (3) surgical approach of EFR or CASL; and (4) pathological maximal diameter of ≤ 1.0 cm. Exclusion criteria: (1) distant metastases exist; (2) other malignant tumors exist; (3) patients with severe psychiatric disorders and uncooperative behavior or multiple organ failure; (4) coagulation disorders or combined bleeding disease; (5) incomplete information. This study has received approval from the institution’s ethics committee, and informed consent has been duly obtained from both the patients and their families. The Medical Ethics Committee of the Liuzhou People’s Hospital affiliated to Guangxi Medical University approved this retrospective cohort study.

### Preoperative Preparation

All patients were evaluated before the procedure, including a review of the patient’s medical history, laboratory tests, computed tomography (CT) scans, electrocardiogram, gastroenteroscopy, and ultrasonography. Tumor size, growth pattern, level of origin, site of growth, internal echoes, and presence of distant metastases were assessed preoperatively. Patients undergoing endoscopic resection were advised to avoid aspirin or other anticoagulant drugs for 1 week. All patients were informed of the risks associated with the procedure and signed a consent form.

### Treatment Process

#### CASL Procedure

**Figure 1.**
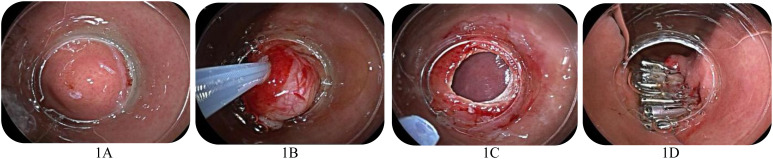
Cap-aspiration lumpectomy for small gastric GIST. A: Endoscopic presentation of small gastric GIST and marking. B: Tightening of the base of the lesion with a wire ring. C: Perforation of the gastric wall. D: Titanium clip to close the defect in the gastric wall.

A transparent cap (D-206-05, Olympus, Japan) was mounted on the distal end of the endoscope (Q26OJ, Olympus, Japan). A bespoke steel wire loop (SD-221U-25, Olympus, Japan) was positioned along the inner rim of the transparent cap, as the internal diameter of the transparent cap used for CASL is about 1.0 cm. Cap-aspiration lumpectomy is suitable for lesions with a diameter of ≤1.0 cm. The lesion was aligned with the cap and drawn towards it. Subsequently, the steel wire loop was affixed at the base of the lesion, and the lesion was excised using high-frequency electricity. The perforation in the gastric wall was sealed with a titanium clip (Figure 1). For extraluminal growths, it is necessary to ensure that the lesion is drawn intact into the transparent cap.

#### EFR Procedure

**Figure 2.**
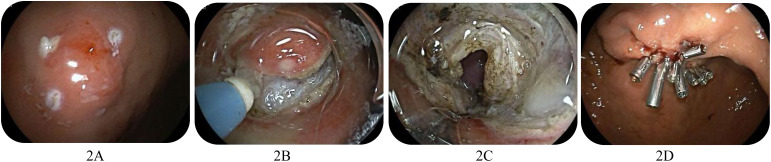
EFR for small gastric GIST. A: Endoscopic presentation of small gastric GIST and marking. B: Peeling off the submucosal layer. C: Perforation of the gastric wall. D: Titanium clip to close the defect in the gastric wall.

All procedures were performed under endotracheal intubation, general anesthesia, and carbon dioxide air pump assistance by an experienced endoscopist. The dual knife (KD 650L, Olympus, Japan) was used to mark the small gastric GIST area. Subsequently, a mixture of saline, methylene blue, and epinephrine was injected into the submucosal layer of the small gastric GIST area. A circumferential incision was made in the small gastric GIST using the dual knife. The submucosal layer was carefully peeled off until the small gastric GIST was completely excised, removing the tumor along with the intrinsic muscularis mucosa and plasma membrane layer. The gastric wall defect was then closed with a titanium clip (Figure 2).

### Pathological Assessment

The specimens were fixed with a 40% formaldehyde solution, sectioned after paraffin embedding, and stained with hematoxylin-eosin. Tumor size, margins, depth of infiltration, and mitosis were recorded. The risk assessment is based on the number of nuclear divisions per 50 high-magnification views and the pathological maximum diameter, size, and location using the National Institutes of Health (NIH) classification.^10^ Tumors were considered to have been completely resected (R0 resection) when the tumor was resected whole (complete resection of the tumor and obtaining a single specimen with no tumor residue on endoscopic view). Furthermore, the lateral and basal margins of the stromal tumor were found to be negative. Immunohistochemical staining was utilized to distinguish GISTs from other tumors of mesenchymal origin. Positivity for CD117 and DOG1 could aid in diagnosing GISTs.

### Complications and Outcome Assessment

Surgical time was defined from entry to exit. The resection time was defined as the interval between the administration of the submucosal injection and the achievement of complete closure of the defect through the utilization of titanium clips. Active hemorrhage was defined as bleeding that compromised the intraoperative visual field, required surgical intervention, and led to a significant reduction in hemoglobin (>2 g/L). Delayed hemorrhage was defined as bleeding caused by postoperative trauma or ulceration. Pneumoperitoneum was defined as the disappearance of hepatic turbidities or the presence of a distinct tympanic sound upon postoperative abdominal percussion. Peritonitis was defined as the presence of pressure, rebound pain, peritoneal tension on abdominal examination, and peritonitis on abdominal CT. The preoperative blood routine was evaluated on the second day following admission, and the postoperative blood routine was evaluated on the second day. Postoperative body temperature was the highest temperature measured within 24 hours after surgery.

### Follow-Up

All patients above were followed up by telephone after 1 month of endoscopic treatment. Additionally, all patients underwent standard endoscopy at months 3, 6, and 12 post surgery to assess for residual tumor or recurrence. Thereafter, endoscopy and abdominal CT scans were repeated annually for evaluation. In addition, imatinib is recommended for intermediate and high-risk patients with close endoscopic monitoring.

### Statistical Methods

SPSS 24.0 software (IBM SPSS Corp.; Armonk, NY, USA) was used for data analysis. Measurement data that conformed to normal distribution were expressed as Mean ± SD, and the comparison between the 2 groups was conducted using an independent samples t-test; those that did not conform to normal distribution were expressed as M (Q1, Q3), and the comparison between the 2 groups was performed using the Mann-Whitney U test. Count data were expressed as cases (%), and comparisons between the 2 groups were made using the chi-square test or Fisher’s exact probability test. *P*<.05 was considered statistically significant.

## Results

### Basic Data and Clinicopathological Characteristics

The present study included a total of 66 patients, comprising 31 males and 35 females. Forty-one patients in the EFR group were 66.0 (63.0, 69.0) years old, while 25 patients in the CASL group were 64.0 (61.0, 68.0) years old. Twenty-one of the 66 patients were hypertensive, 7 were diabetic, and 5 patients had both hypertension and diabetes; 51 tumors were located in the fundus (77.3%), 11 in the body (16.7%), 3 in the sinus (4.5%), and 1 in the cardia (1.5%); the tumor was observed using ultrasonographic endoscopy. The tumors were located in the fundus of the stomach in 51 cases (77.3%), the body of the stomach in 11 cases (16.7%), the gastric sinus in 3 cases (4.5%), and the cardia in 1 case (1.5%). Ultrasonographic endoscopic observation revealed that the tumors in a total of 62 cases (93.9%) had originated in the intrinsic muscularis layer, while only 4 cases (6.1%) had originated in the submucosal layer. Furthermore, 52 cases (78.8%) exhibited intraluminal growth, 5 cases (7.6%) had extra-luminal growth, and 9 cases (13.6%) showed both intra- and extra-luminal growth.

With regard to the variables of gender, age, comorbidities, tumor location, and growth pattern, postoperative pathology showed that the maximum diameter of the tumor in the EFR group was larger than that in the CASL group, and the difference between the 2 groups was statistically significant (*P* = .01). Sixty-six cases were very low risk according to NIH risk classification of postoperative pathology (Table 1).

### Comparison of Surgical Conditions

All lesions were resected whole without tumor rupture, intraoperative active bleeding, or referral to surgery. The incidence of pneumoperitoneum was 3 cases in the EFR group and 2 cases in the CASL group, and the difference was not statistically significant (*P* = 1.00). Intraoperative hot-clamp hemostasis was performed in 31 patients in the EFR group, whereas only 3 instances of hot-clamp hemostasis were observed in the CASL group. The observed difference was statistically significant (*P* < .01). The number of titanium clamp sutures was 6.8 ± 3.2 in the EFR group and 5.2 ± 2.0 in the CASL group, with a statistically significant difference (*P* = .03). The operative and resection times in the CASL group were found to be significantly shorter than those in the EFR group. The R0 resection rate in the EFR group was 95.1%, with 2 cases of incomplete resection under the microscope. In comparison, the R0 resection rate in the CASL group was 100.0%, with no statistically significant difference (*P* = .52) (Table 2).

### Comparison of Postoperative Efficacy

The postoperative white blood cell count (*P* = .01) and neutrophil percentage (*P* = .03) were observed to be higher in the EFR group than in the CASL group. Postoperative antibiotics were used in 31 cases in the EFR group and in 6 cases in the CASL group, and the observed difference was found to be statistically significant (*P* < .01). The mean number of days of postoperative antibiotics administered to patients in the EFR group was 2.8 ± 2.0, while the mean number of days of postoperative antibiotics administered to patients in the CASL group was 1.0 ± 2.0. This difference was statistically significant (*P* = .01). More patients in the EFR group had postoperative fever compared to the CASL group (*P* = .01). Sixty-six patients did not exhibit any symptoms. In the study, the difference was statistically significant (*P* = .01). There were more patients with postoperative fever in the EFR group than in the CASL group (*P* = .01). None of the 66 patients suffered from postoperative peritonitis or delayed hemorrhage. The interval until the first postoperative administration of fluids was longer in the EFR group than in the CASL group (*P* = .05). No significant difference was observed in the postoperative length of stay and total hospital days (Table 3).

### Hospitalization Costs and Follow-Up

The mean total hospitalization cost was higher in the EFR group than in the CASL group (¥13 595.0 ± 2653.3) versus (¥11 209.0 ± 2458.9), *t* = 7.32, *P* < .01, with a cost saving of 35.9% in CASL. The principal factor contributing to the discrepancy in cost between the EFR and CASL groups is the expense associated with the materials utilized. The dual knife (KD 650L, Olympus, Japan) is approximately ¥2000 more costly than the transparent cap (D-206-05, Olympus, Japan) and the bespoke steel wire loop (SD-221U-25, Olympus, Japan). Additional minor expenses are incurred as a result of hospitalization. The follow-up period for the EFR group was 18.2 ± 6.2 months, while the CASL group had a follow-up period of 17.3 ± 5.3 months. During the follow-up period, both groups of patients remained alive and exhibited no evidence of tumor recurrence or metastasis. However, the median follow-up time was inadequate in the present study. Subsequent studies will seek to collect long-term follow-up data to analyze the long-term efficacy of the treatment and the risk of relapse.

## Discussion

Gastric small stromal tumors are most common in the middle-aged and elderly population. The incidence is increasing among the elderly, with some studies reporting a detection rate of up to 22.5% at autopsy in patients over 50 years old.^2^ The median age of patients with gastric GISTs in previous studies was mostly under 60 years old. There is a relative scarcity of studies examining potential differences in the clinical characteristics, treatment, and prognosis of gastric stromal tumor patients aged ≥60 years and older. There is also no consensus on the management of small gastric GISTs in elderly patients among different guidelines.

Previous studies have found that the prognosis of GISTs is age-related. Elderly patients with GISTs tend to have a poorer prognosis and may require a different treatment approach compared to younger patients. This is due to the gradual decline in organ function, higher prevalence of underlying diseases, and poorer treatment adherence in the elderly population. Elderly patients have high surgical risks and high rates of postoperative complications. Endoscopic surgery offers several advantages over traditional surgical techniques. These include minimal invasiveness, a reduced incidence of complications, and a lower cost.^8^^,^^11^^,^^12^ Endoscopic surgical modalities include endoscopic submucosal removal, EFR, endoscopic submucosal dissection (ESD), and endoscopic mucosal resection, and EFR is more suitable for treating gastric GISTs originating from the intrinsic muscularis propria layer or closely connected to the submucosal layer compared to other endoscopic procedures.^13^^,^^14^ In order to achieve R0 resection, gastric GISTs that are adherent to the gastric serosa layer must be incised through the entire layer of the gastric wall in order to create an iatrogenic perforation. Once the resection is complete, the incision in the gastric wall must be closed with titanium clips. A substantial body of evidence attests to the safety, simplicity, and feasibility of EFR as a method for the removal of gastric GISTs originating from the muscularis propria.^15^^,^^16^ As a modified procedure of EFR, CASL has also become commonly used for the resection of small gastric GISTs. Cap-aspiration lumpectomy is an effective and minimally invasive treatment for tumors, with a high rate of complete and rapid tumor removal, minimal adverse events, and a low incidence of associated perforations that can be readily repaired endoscopically.^17^^,^^18^ Compared to other endoscopic techniques, CASL is more time-efficient, straightforward to manage postoperatively, and does not necessitate the use of specialized endoscopic equipment. A paucity of literature exists concerning the comparative efficacy and safety of EFR and CASL for the treatment of small gastric GIST, particularly in elderly patients.

The study population consisted of patients aged 60 years and above, with a pathological maximum diameter of the small gastric GIST of no greater than 1.0 cm. As a consequence of the restricted internal diameter of the hyaline cap, the size of the small gastric GISTs in the EFR group was marginally larger than that observed in the CASL group. Based on clinical practice experience, the success rate and complete resection rate of CASL surgery for stromal tumors larger than 1.0 cm is low. It is worth noting that, as stated by Meier et al,^13^ not only the size of the tumor but also the characteristics of the tumor, such as tumor location, and intraluminal or extraluminal growth, determine the likelihood of complete resection. Therefore, a thorough preoperative evaluation should be conducted to select the most suitable surgical approach. Previous studies confirmed that the R0 resection rate of endoscopic treatment for gastric GISTs (≤ 3 cm) in elderly patients was 95.7%.^19^ The findings of the present study indicate that both EFR and CASL are effective in achieving complete resection rates in the treatment of small gastric GISTs in elderly patients, with rates of 95.1% and 100.0%, respectively. The 2 incompletely resected lesions were located in the gastric fundus and cardia, respectively. They were both endoscopically bulging lesions with smooth surfaces and ultrasonographic endoscopic evidence of intraluminal growths originating in the intrinsic muscularis layer. Both lesions were classified as very low risk, and neither of them recurred or metastasized during the follow-up period. The reason for the lower R0 resection rate of EFR compared to CASL is the possibility of mechanical damage to the tumor envelope during resection along the muscularis propria, resulting in residual tumor cells under the microscope.

On the other hand, for the resection of small gastric GISTs in elderly patients, although both surgical methods had shorter operation times and fewer intraoperative and postoperative complications, CASL held a significant advantage in both total operation time and lesion resection time. The use of thermal hemostatic forceps was also less, reducing the time of contact between the abdominal cavity and the gastric cavity, and lowering the risk of abdominal infection. In addition, the postoperative inflammatory response and antibiotic use were significantly higher in EFR patients than in CASL patients. Postoperative recovery of gastric function was faster in both groups, with no significant difference in the postoperative recovery between the 2 groups. The postoperative recovery of gastrointestinal function was faster in both groups, with no significant difference between the CASL group and the control group. The CASL group spent significantly less on hospitalization than the EFR group, with the CASL group saving nearly one-sixth of the cost. The common complications of endoscopic surgery are bleeding and perforation;EFR and CASL are prone to gastrointestinal perforation during the operation in order to achieve complete resection.

Gastric GISTs are predominantly situated in the fundus of the stomach, which is characterized by a dense network of vascular structures. Following the CASL procedure, it is crucial to utilize the transparent cap and injection pump to accurately identify the source of bleeding and employ the heat clamp to promptly halt the bleeding. This approach can effectively address the bleeding vessels at the lesion, ensuring a safe and effective treatment outcome. Adequate suction is employed to minimize trauma, and chain sutures are performed using titanium clips, commencing from one side of the trauma. This allows for effective clamping and closure of the trauma.

As the over-the-scope clip (OTSC) release device is a disposable device, the anastomotic clip cannot be relocated once it has been positioned. Consequently, the only available options are to add metal clips locally or to remove the current anastomotic clip. Furthermore, the cost of the OTSC is considerable, and there are certain limitations in terms of economic benefits. The OTSC has been demonstrated to be a more effective method of preventing perforation and reducing complications.^20^^-^^22^ It is capable of grasping a larger surface area and achieving a higher closure force, which allows it to reach deeper layers of tissue.^23^ The utilization of an over-the-scope clip for the management of EFR represents a safe alternative to potentially morbid operative intervention. The CASL procedure can be performed using OTSC to prevent bleeding and perforation. The OTSC facilitates the closure of full-layer defects in a relatively brief time frame with a relatively uncomplicated procedure. The success rate for this is 100%, with a notably lower incidence of complications.

Clinical studies have demonstrated that OTSC and conventional titanium clip suturing of upper gastrointestinal bleeding wounds exhibited a hemostasis success rate of 98.4% in the OTSC group and 78.4% in the conventional titanium clip group (*P* = .001).^24^ The incidence of perforation in ESD using conventional titanium clips was 0.13%, while the incidence of perforation was 0% using OTSC to clip the wound.^23^^,^^25^Therefore, the OTSC can effectively reduce the risk of bleeding and perforation. It can be reasonably deduced that OTSC has potential applications in future studies.

Adequate suction is employed to minimize trauma, and chain sutures are performed using titanium clips, commencing from one side of the trauma. This approach is intended to minimize trauma and facilitate effective closure of the injury. This enables effective clamping and closure of the trauma. In this study, all lesions underwent iatrogenic perforation in order to achieve an R0 resection rate. However, the perforated wound could be readily visualized and repaired endoscopically with titanium clips without an increase in serious procedure-related adverse events. The majority of published studies have also demonstrated that iatrogenic perforations can be effectively managed through endoscopic techniques.^26^^,^^27^ The perioperative adverse events of endoscopic treatment were all controllable because the lesions were all small. The preceding study demonstrated that the EFR of gastric GISTs (≤5 cm) was safe and cost-effective and exhibited superior perioperative outcomes and lower costs compared with laparoscopic or surgical resection.^28^ But intraluminal gastric GISTs and those located at the esophagogastric junction or near the pylorus are difficult to resect by EFR.^29^ EFR procedure can be performed by collaborating with surgery as a multidisciplinary treatment and endoscopic full-thickness gastric resection.

Both the EFR and CASL are safe and feasible for the endoscopic resection of small gastric GISTs in the elderly. Nonetheless, research comparing the outcomes of EFR and CASL is sparse. Cap-aspiration lumpectomy is more suitable for treating small gastric GISTs in elderly patients due to shorter operative time, economic advantages, and reduced risk of surgical infection.

The present study was a single-center retrospective study in which lesions were all ≤1.0 cm in size. Additionally, and all operations were performed by experienced endoscopists. Since most small gastric GISTs are located in the gastric fundus, it is unclear whether CASL is feasible for cardia and sinus lesions.

There are several limitations to the research study. First, this was a single-center and retrospective study. Hence, there might have been some potential selection bias. Second, the median follow-up time was not long enough in this study. With further follow-up, the results will be more accurate and credible. Third, the sample size was relatively small, with only 66 cases. The discrepancy in sample sizes between the CASL group (n = 25) and the EFR group (n = 41) may reduce the reliability of statistical comparisons. Thus, large-scale data could improve the robustness of the analysis.

In conclusion, the management treatment of small gastric tumors in elderly patients with existing conditions should consider the preferences and opinions of the patients and their families. A personalized, individualized, and multidisciplinary approach should implemented. This study contributes to enhancing the safety and efficacy of endoscopic treatment for small gastric stromal tumors (≤ 1.0 cm) in elderly patients. The findings suggest that CASL may be a more suitable treatment option for small gastric GISTs located in the gastric fundus and body in elderly patients. This is due to the shorter operative time, reduced postoperative inflammation, and lower medical costs associated with CASL compared to EFR. Nevertheless, further research is required in the form of large-sample, multicenter, prospective studies to validate the results and to further define the indications for CASL.

## Figures and Tables

**Table 1.** Comparison of Basic Data and Clinic Characteristics of Elderly Patients with Small Gastric Gastrointestinal Stromal Tumors in Different Groups

Clinical Information	EFR Group (n = 41)	CASL Group (n = 25)	Statistic	*P*
Sex (M/F)	19/22	12/13	*χ*^2^ = 0.02	.89
Age [years, median (Q1,Q3)]	66.0 (63.0,69.0)	64.0 (61.0,68.0)	*U* = 396.00	.12
Complications (%)	20 (48.8)	13 (52.0)	*χ*^2^ = 0.06	.80
Preoperative white blood cell count (×109/L, Mean ± SD)	5.1 ± 1.1	4.6 ± 1.3	*t* = 1.69	.09
Preoperative neutrophil percentage (%)	56.6 ± 7.9	53.6 ± 8.0	*t* = 1.48	.14
Tumour site (%)				.49^a^
Cardia	1 (2.4)	0 (0)		
Gastric fundus	31 (75.6)	20 (80.0)		
Gastric body	6 (14.6)	5 (20.0)		
Gastric antrum	3 (7.3)	0 (0)		
Level of origin (%)				.02^a^
Submucosa	0 (0)	4 (16.0)		
Intrinsic muscle layer	41 (100.0)	21 (84.0)		
Tumour growth pattern (%)				.81^a^
Intratumoral growth	31 (75.6)	21 (84.0)		
Extracavitary growth	4 (9.8)	1 (4.0)		
Intratumoral and extra-cavitary growth	6 (14.6)	3 (12.0)		
Maximum diameter of tumor (Mean ± SD)	1.1 ± 0.3	0.9 ± 0.3	*t* = 3.42	.01
Nuclear schizophrenia [d/50 HPF, M (Q1,Q3)]	1.0 (0.5,3.3)	1.0 (0.0,2.5)	*U* = 453.00	.43
NIH Risk Classification: Very Low Risk (%)	41 (100)	25 (100.0)	–	–

‘-’ means that no statistical test was performed.

CASL, cap-aspiration lumpectomy; EFR, endoscopic full thickness resection group.

^a^Use of Fisher’s exact probability method by the Service.

**Table 2.** Comparison of Surgery in Elderly Patients with Small Gastric Gastrointestinal Stromal Tumors in Different Groups

Observation Indicators	EFR Group (n = 41)	CASL Group (n = 25)	Statistic	*P*
Surgical time [min, median (Q1,Q3)]	45.0 (32.5,66.5)	30.0 (20.0,42.5)	*U* = 259.50	.01
Excision time [min, median (Q1,Q3)]	30.0 (20.0,50.5)	9.0 (6.5,16.5)	*U* = 127.00	<.01
Lumpectomy (%)	41 (100.0)	25 (100.0)		1.00^a^
Titanium clamp suture (%)	41 (100.0)	25 (100.0)		1.00^a^
Number of titanium clips (Mean±SD)	6.8 ± 3.2	5.2 ± 2.0	*t* = 2.20	.03
Pneumoperitoneum (%)	3 (7.3)	2(8.0)		1.00^a^
Thermal forceps for haemostasis (%)	31 (75.6)	3 (12.0)	*χ*^2^ = 25.16	<.01
R0 polypectomy (%)	39 (95.1)	25 (100.0)		.52^a^

CASL, cap-aspiration lumpectomy; EFR, endoscopic full thickness resection group.

^a^Use of Fisher’s exact probability method by the service.

**Table 3.** Comparison of Postoperative Outcomes of Elderly Patients with Small Gastric Gastrointestinal Stromal Tumors in Different Groups

Observation Indicators	EFR Group (n = 41)	CASL Group (n = 25)	Statistic	*P*
Postoperative white blood cell count [×10^9^/L, median (Q1,Q3)]	8.3 (6.6, 10.4)	6.3 (5.0, 7.7)	*U* = 271.00	.01
Postoperative neutrophil percentage (%, Mean ± SD)	77.6 ± 8.8	73.0 ± 6.8	*t* = 2.25	.03
Antibiotic use (%)	31 (75.6)	6(24.0)	*χ*^2^ = 16.79	<.01
Days of postoperative antibiotic use (day, Mean ± SD)	2.8 ± 2.0	1.0 ± 2.0	*t* = 3.63	.01
Postoperative fever (%)	23 (56.1)	6 (24.0)	*χ*^2^ = 6.49	.01
First time eating [d, M(Q1,Q3)]	2.0 (1.5, 3.0)	1.0 (1.0, 2.5)	*U* = 370.50	.05
Post-operative length of stay (d, Mean ± SD)	3.7 ± 1.5	3.1 ± 1.6	*t* = 1.55	.13
Total days of hospitalization (d, Mean ± SD)	7.1 ± 2.0	6.2 ± 2.2	*t* = 1.69	.09

CASL, cap-aspiration lumpectomy; EFR, endoscopic full thickness resection group.

## Data Availability

The data that support the findings of this study are available on request from the corresponding author.
